# Marine Collagen Matrix Carrier with Injectable Platelet-Rich Fibrin in Management of Gingival Recession Defects

**DOI:** 10.1155/2023/3357323

**Published:** 2023-12-06

**Authors:** Deepikha Krishnaraj, Harinath Parthasarathy, Anupama Tadepalli, Deepa Ponnaaiyan, A. Thirumal Raj, Shankargouda Patil, Snehashish Ghosh

**Affiliations:** ^1^Department of Periodontics, SRM Dental College and Hospital, Bharathi Salai, Ramapuram, Chennai 600089, Tamil Nadu, India; ^2^Dept. of Oral Pathology and Microbiology, Sri Venkateshwara Dental College and Hospital, Chennai, India; ^3^College of Dental Medicine, Roseman University of Health Sciences, South Jordan, Utah-84095, USA; ^4^Centre of Molecular Medicine and Diagnostics (COMManD), Saveetha Dental College and Hospitals, Saveetha Institute of Medical and Technical Sciences, Saveetha University, Chennai, India; ^5^Department of Oral Pathology, College of Medical Sciences, Bharatpur, Nepal

## Abstract

**Background:**

The gold standard in the management of gingival recession (GR) defects has been connective tissue graft (CTG) with coronally advanced flap (CAF). But patient morbidity associated with graft harvesting is a major drawback and has led to the development of various substitute biomaterials which have been tried and tested.

**Aim:**

Our study is aimed at checking the efficacy of marine collagen matrix (MCM) impregnated with injectable platelet-rich fibrin (I-PRF) with modified CAF in the treatment of Miller's class I and II recession defects. *Case Description.* Six patients with ten GR defects in maxilla were treated with CAF + MCM + I-PRF. Clinical parameters like recession height (RH), recession width (RW), root coverage (RC%), width of attached gingiva (WAG), keratinized tissue height (KTH), probing pocket depth (PPD), clinical attachment level (CAL), gingival biotype (GB), plaque index (PI), and visual analogue score (VAS-E) esthetic scores were evaluated up to six months. There was significant root coverage observed at three- and six-month follow-ups.

**Conclusion:**

The proposed treatment was effective in the management of GR defects and improvement in soft tissue biotype without the morbidity associated with soft tissue harvest.

## 1. Introduction

Gingival recession defects are ubiquitous in all populations and present with them several negative functional and esthetic sequelae [1]. The gold standard treatment for managing recession defects is coronally advanced flap (CAF) with connective tissue graft (CTG) [2] that has demonstrated the most predictable outcomes, long-term stability, [3, 4], and good esthetics [5]. Major limitations of using CTG are the involvement of a second surgical site that increases patient morbidity, problems with the availability of graft tissue when treating multiple recession defects, or when patients have thin biotype and increased surgical time. To overcome these limitations, various soft tissue graft substitutes have been researched that could deliver comparable results to the gold standard. Here, we use marine collagen matrix (MCM) infused with injectable platelet-rich fibrin (I-PRF) as a graft substitute along with CAF.

Collagen is an eminent protein of the extracellular matrix, and as a biological scaffold, it facilitates vascular growth [6] via the directed migration of cells and aids in the deposition of oriented and organized fibers that increase the integrity of the tissue [7]. Collagen from marine sources has structural similarity and better physiologic and biochemical properties in comparison to collagen obtained from terrestrial sources without the risk of zoonotic diseases [8, 9]. Autologous platelet concentrates are used in various fields of medicine and surgery for decades for its property to expedite wound healing [10]. I-PRF, a liquid type of platelet-rich fibrin (II generation platelet concentrate) proposed by Mourao et al. [11], demonstrated additional advantages to conventional PRF. I-PRF could increase fibroblast migration, release increased levels of growth factors [12], enhance angiogenic activity, upregulate wound healing [13], and also increase osteoblast migration, adhesion, proliferation, and differentiation [14]. It also exhibited considerable antibacterial activity against periodontal pathogens [15].

Bovine/porcine collagen matrices (CM) and autologous platelet concentrates have been used separately in the treatment of recession defects as biological substitutes with good results [16–21], but there is insufficient evidence to prove its combined efficacy in recession management. Thereby, we hypothesize that MCM, along with the mentioned advantages, can be used as a carrier for I-PRF for its containment at the defect site and, together, could have synergistic activity to expedite wound healing and regeneration when used in the management of gingival recession defects.

## 2. Case Description

Six patients with ten gingival recession defects in maxilla were included in this case series (Table 1). All patients gave their consent in writing and verbally.

### 2.1. Presurgical Phase

The presurgical phase consisted of phase I periodontal therapy, documentation, and evaluation of clinical parameters such as recession height (RH), recession width (RW), probing pocket depth (PPD), clinical attachment level (CAL), width of attached gingiva (WAG), keratinized tissue height (KTH), gingival biotype (GB), and plaque index (PI) (Table 2). Customized stents were used for standardizing the values.

### 2.2. I-PRF Protocol

**Figure 1:**
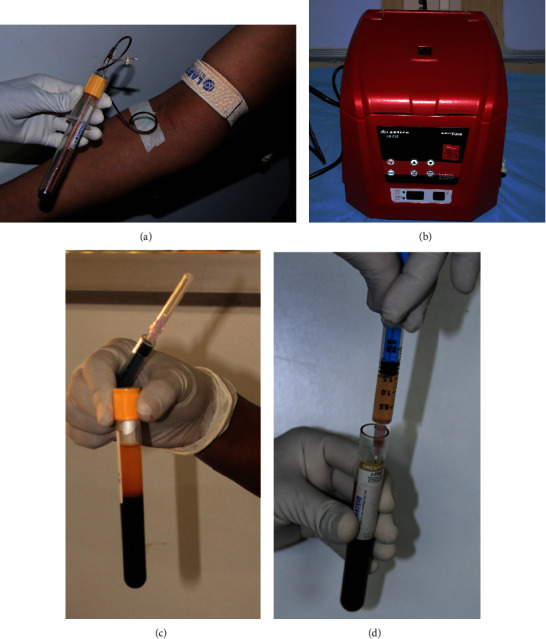
I-PRF protocol. (a) Collection of blood from anticubital vein in specified vacutainers. (b) Centrifugation done at 700 rpm for 3 minutes at 60 G force with Dentifuge LD C-10. (c) I-PRF separated as upper liquid yellow phase. (d) I-PRF retrieved with 2 ml syringe.

10 ml of whole blood was drawn from the subjects from the anticubital vein and was collected in manufacturer-specified I-PRF tubes without any additives. It was centrifuged at room temperature in a preprogrammed centrifuge (Dentifuge LD C-10^®^ Labtech Disposables, Ahmedabad 380015, Gujarat, India) at a predetermined spin for I-PRF (700 rpm with 60 g force for 3 minutes). After centrifugation was complete, the blood split into a yellow-orange upper phase and a red lower phase. The upper phase which was the liquid PRF was then retrieved using a sterile syringe (Figure 1). I-PRF obtained was then used to impregnate MCM (Biofil sponge^®^ Eucare Pharmaceuticals, Thirumudivakkam, Chennai 600044, Tamil Nadu, India).

### 2.3. Surgical Phase

**Figure 2:**
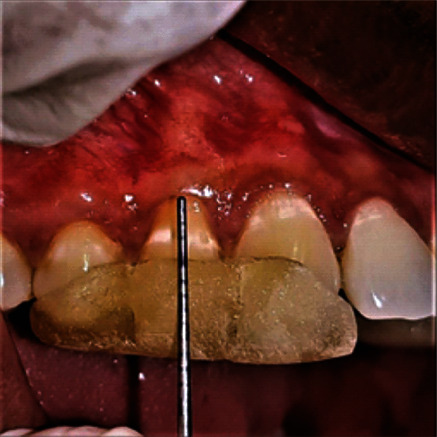
Baseline (patient: 1).

**Figure 3:**
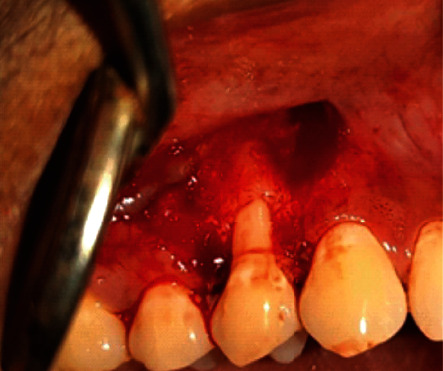
Flap design (patient: 1).

**Figure 4:**
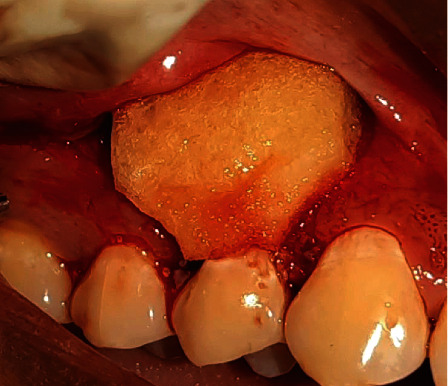
Adaptation of collagen sponge impregnated with I-PRF over the defect site (patient: 1).

**Figure 5:**
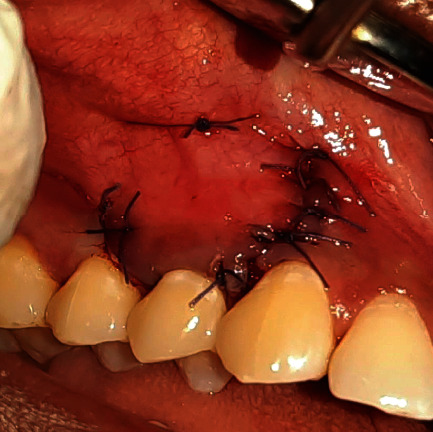
Approximation of the flap and suturing (patient: 1).

Under local anesthesia (2% Lignocaine, 1 : 80,000 adrenaline), surgical papilla was created by giving a v-shaped beveled incision on the mesial and distal aspects of the recession defect of the interdental papilla, with a modification of de Sanctis and Zucchelli's CAF technique [22] which would allow a better adaptation of flap over the deepithelialized papillary bed. At the end of these incisions, two vertical, slightly divergent incisions were created and continued up to the alveolar mucosa to approximate the flap without tension. A split-full split method was used to elevate the resulting trapezoidal flap. Residual interdental papilla was deepithelialized. Root surface debridement was done. The MCM was trimmed according to the defect contour and was impregnated with I-PRF which takes around 10-12 minutes to polymerize, and it was then adapted over the root surface and flap advanced slightly coronal to cementoenamel junction and secured using 4-0 Vicryl sling sutures. Simple interrupted sutures were used to approximate vertical releasing incisions. The flap's base was secured with periosteal anchoring sutures (Figures 2–5).

**Figure 6:**
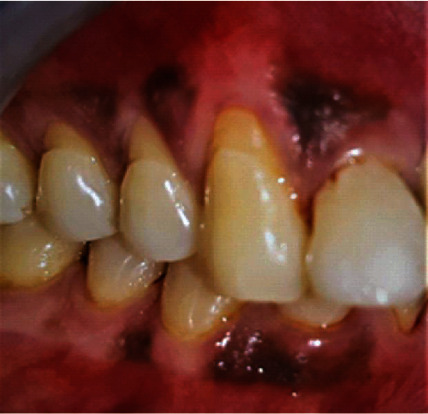
Baseline (patient: 6).

**Figure 7:**
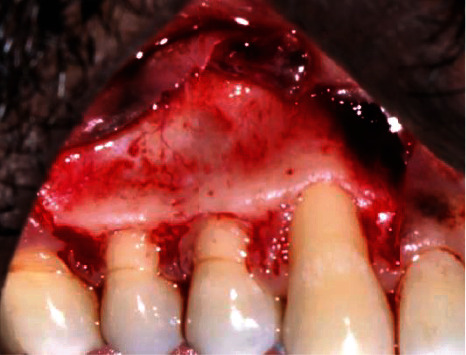
Flap design (patient: 6).

**Figure 8:**
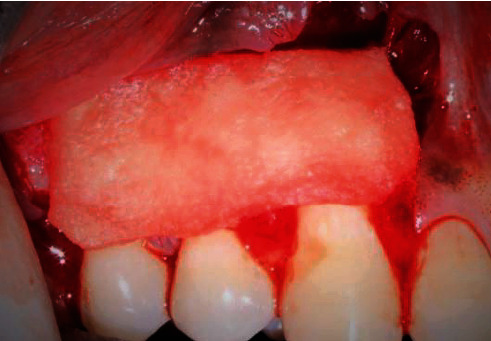
Adaptation of collagen sponge impregnated with I-PRF over the defect site (patient: 6).

**Figure 9:**
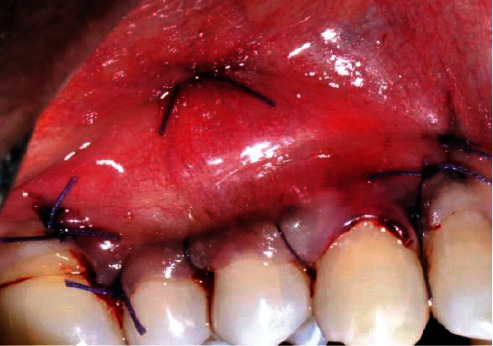
Approximation of the flap and suturing (patient: 6).

In multiple recession defects, similar procedure was followed. V-shaped beveled incision was placed on the mesial and distal papilla on either side of the teeth involved to create surgical papilla followed by vertical divergent incisions. A split-full split approach to elevate the flap was done to raise the flap. Residual interdental papilla was deepithelialized followed by root surface debridement. The MCM was trimmed according to the defect contour and was impregnated with I-PRF, and it was then adapted over the root surface and flap advanced slightly coronal to cementoenamel junction and secured using continuous sling sutures. Simple interrupted sutures were used to approximate vertical releasing incisions. The flap's base was secured with periosteal anchoring sutures (Figures 6–9).

Patients were prescribed antibiotics and analgesics for five days. For four weeks, patients were advised to use chlorhexidine mouthwash and avoid brushing the surgical site. Sutures were removed two weeks postsurgery.

### 2.4. Statistical Analysis

All statistical analysis was performed using SPSS Software (IBM SPSS Statistics for Windows, version 26.0). To compare values between time points (baseline, 1 month, 3 months, and 6 months), Friedman's test for repeated measures was used followed by the Bonferroni adjusted Wilcoxon signed-rank test for multiple pairwise comparison. Mean (M) and standard deviation (SD) were used to express the data. Significance level was fixed at 5% (*α* = 0.05). *p* value of < 0.05 was considered statistically significant.

## 3. Results

**Figure 10:**
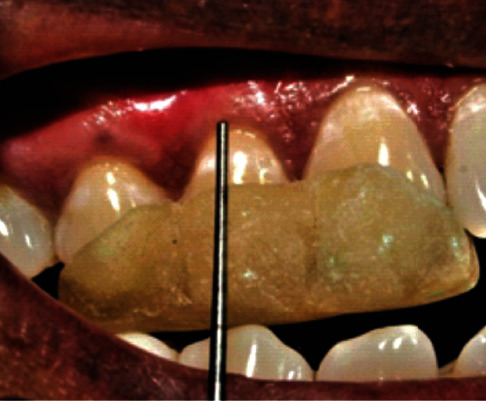
Follow-up at 6 months (patient: 1).

**Figure 11:**
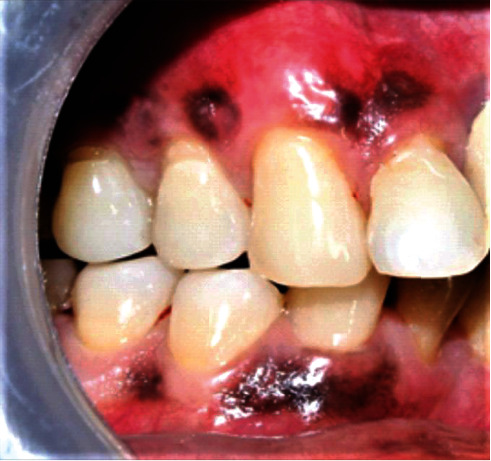
Follow-up at 6 months (patient: 6).

**Figure 12:**
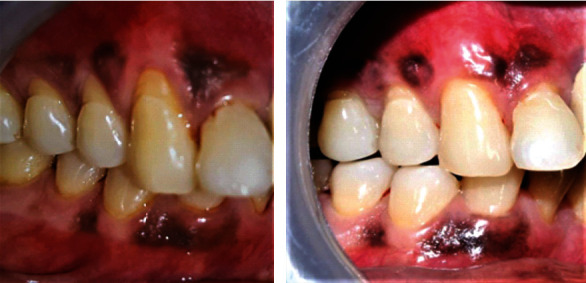
Baseline vs. 6 months postoperative photographs (patient: 6).

All the patients exhibited satisfactory healing. There was significant improvement noted in all parameters at 6 months (Figures 10, 11, and 12) (Tables 3 and 4). Four sites showed complete root coverage (CRC), four sites with thin biotype at baseline exhibited thick biotype, width of attached gingiva (WAG), and keratinized tissue height (KTH) showed a slight increase at six months postsurgery. There were no postoperative complications.

## 4. Discussion

Though PRF and CM have been used in the management of gingival recession defects separately, the literature lacks evidence of the MCM + I-PRF combination being used to treat recession defects. So, comparisons of results could not be made with available literature. Mean gain in recession depth was 1.7 mm, and a mean root coverage percentage (RC %) of 71.7 ± 24.91% was achieved with significant CAL gain at six months. Two sites that showed CRC at 3 months showed 50% root coverage (RC) at 6 months which might be attributable to the volumetric shrinkage of the collagen matrix [17]. One site with glass ionomer cement (GIC) filling showed 50% coverage, at 3 and 6 months which is contradictory to literature that showed successful outcomes when root coverage surgery was performed on the restored root surfaces [23].

Owing to the improvement in gingival thickness, the technique and biomaterials used in the current study could be a viable alternative to improve gingival biotype (GB). Improvement in gingival thickness following recession management was also noted in studies done by Aroca et al. [24], Cardarapoli et al. [25] who used collagen scaffolds, and Gupta et al. [20] and Eren and Atilla [26] who used PRF membranes. Plaque index (PI) showed significant improvement at 1 and 3 months, but there was a slight decline at 6 months due to the inability to perform professional oral hygiene procedures due to the corona pandemic. Two out of three sites that showed increased PI had 50% relapse, emphasizing the critical role of hygiene maintenance for the stability of achieved results. [27] Visual analogue scale-esthetics (VAS-E) scores that rated patient's perception towards the treatment outcome decreased from 9.1 ± 0.99 at 3 months to 8.9 ± 0.99 at 6 months owing to relapse at 2 sites, but overall, there was a satisfactory patient outcome.

I-PRF-infused MCM could be an affordable and potential alternate to CTG, thereby avoiding soft tissue harvest and its associated morbidity/limitations. I-PRF could be used to enhance the bioactivity of other graft materials, while MCM could be used as a carrier for growth factors and drugs that could be used for healing and regenerative purposes. The limitations of the study include a small sample size, shorter follow-up period, and absence of a control group which might limit the generalizability of the findings.

## 5. Conclusion

With the cumulative observations made in the present study, it can be proposed that I-PRF-impregnated MCM as a bioactive carrier might be capable of enhancing soft tissue regenerative outcomes with specific indications such as root coverage procedures. Owing to the study's limitations, additional research with larger sample sizes and extended follow-up periods is required to ensure the external validity of the study findings and also to explore and exploit the synergistic effects of I-PRF + MCM in various domains of periodontal plastic surgery.

## Figures and Tables

**Table 1:** Study participants' demographics.

		Number of sites
Gender	Male	8
	Female	2

Class of recession	Class I	9
	Class II	1

Biotype baseline	Thin	4
	Thick	6

Teeth sites involved	Centrals	1
	Laterals	1
	Canines	2
	1^st^ premolars	5
	2^nd^ premolars	1

**(a):** 

No.	Pt. age/gender	Tooth no.	RH			RW			RC%		PI		
			B	3 M	6 M	B	3 M	6 M	3 M	6 M	B	3 M	6 M
1	33/f	24	2	0	0	4	0	0	100%	100%	1	.5	.5
2	54/m	14	2	1	1	3	3	3	50%	50%	1.5	.5	.5
3	44/f	24	2	0	1	4	0	3	100%	50%	.5	0	0
4	35/m	14	2	0	1	3	0	2	100%	50%	1	0	.5
5	30/m	21	4	2	2	5	4	4	50%	50%	1.5	.5	1
		22	2	1	1	4	3	3	50%	50%	1.5	.5	.5
		23	2	0	0	4	0	0	100%	100%	1.5	.5	.5
6	52/m	13	3	0	0	5	0	0	100%	100%	.5	0	0
		14	3	1	1	5	3	3	66.6%	66.6%	.5	0	0
		15	2	0	0	4	0	0	100%	100%	1	0	.5

RH: recession height; RW: recession width; RC%: root coverage percentage; PI: plaque index.

**(b):** 

No.	Pt. age/gender	Tooth no.	PPD			CAL			WAG			KTH		
			B	3 M	6 M	B	3 M	6 M	B	3 M	6 M	B	3 M	6 M
1	33/f	24	1	1	1	3	1	1	1	1	2	2	2	3
2	54/m	14	1	1	1	3	2	2	1	1	2	2	2	3
3	44/f	24	1	1	1	3	1	2	1	2	2	2	3	3
4	35/m	14	1	1	1	3	1	2	1	1	1	2	2	2
5	30/m	21	1	1	1	5	3	3	1	1	1	2	2	2
		22	1	1	1	3	2	2	2	2	2	3	3	3
		23	1	1	1	3	1	1	1	2	2	2	3	3
6	52/m	13	1	1	1	4	1	1	1	2	2	2	3	3
		14	1	1	1	4	2	2	1	1	1	2	2	2
		15	2	2	2	4	2	2	1	1	1	3	3	3

PPD: probing pocket depth; CAL: clinical attachment level; WAG: width of attached gingiva; KTH: keratinized tissue height.

**(c):** 

No.	Pt. age/gender	Tooth no.	GB		VAS-E	
			B	6 M	3 M	6 M
1	33/f	24	Thin	Thick	10	10
2	54/m	14	Thick	Thick	8	8
3	44/f	24	Thick	Thick	10	8
4	35/m	14	Thick	Thick	8	8
5	30/m	21	Thick	Thick	8	8
		22	Thick	Thick	8	8
		23	Thick	Thick	10	10
6	52/m	13	Thin	Thick	10	10
		14	Thin	Thick	9	9
		15	Thin	Thick	10	10

GB: gingival biotype; VAS-E: visual analogue scale-esthetics.

**Table 3:** Study parameters at various time points.

Parameters	Baseline (mean ± S.D)	1 month (mean ± S.D)	3 months (mean ± S.D)	6 months (mean ± S.D)
RH (mm)	2.4 ± 0.7	0.3 ± 0.48	0.5 ± 0.71	0.7 ± 0.67
RW (mm)	4.1 ± 0.74	0.9 ± 1.45	1.3 ± 1.70	1.8 ± 1.62
RC%	NA	NA	81.7 ± 24.16	71.7 ± 24.91
PPD (mm)	1.1 ± 0.32	NA	1.1 ± 0.32	1.1 ± 0.32
CAL (mm)	3.5 ± 0.71	NA	1.6 ± 0.7	1.8 ± 0.63
KTH (mm)	2.2 ± 0.42	2.5 ± 0.53	2.5 ± 0.53	2.7 ± 0.48
PI	1.1 ± 0.44	0.4 ± 0.24	0.3 ± 0.26	0.4 ± 0.32
GB				
6 (60%)	Thick	NA	NA	10 (100%)
4 (40%)	Thin			
VAS-E	NA	NA	9.1 ± 0.99	8.9 ± 0.99

RH: recession height; RW: recession width; RC%: root coverage percentage; PPD: probing pocket depth; CAL: clinical attachment level; KTH: keratinized tissue height; PI: plaque index; GB: gingival biotype; VAS-E: visual analogue scale-esthetics.

**Table 4:** Variations of parameters across the time intervals.

Parameters	Baseline vs. 3 months (*p* value)	Baseline vs. 6 months (*p* value)	3 months vs. 6 months (*p* value)
RH	0.003^∗∗^	0.019^∗∗^	1.00
RW	0.011^∗∗^	0.056	1.00
RC%	NA	NA	0.157
PPD	—	—	1.00
CAL	0.001^∗∗∗^	0.005^∗∗^	1.00
KTH	1.00	0.500	1.00
PI	0.001^∗∗∗^	0.011^∗∗^	1.00

RH: recession height; RW: recession width; RC%: root coverage percentage; PPD: probing pocket depth; CAL: clinical attachment level; KTH: keratinized tissue height; PI: plaque index. *p* ≤ 0.001^∗∗∗^: 99.9% significant; *p* ≤ 0.01^∗∗^: 99%; and *p* ≤ 0.05^∗^: 95% significant.

## Data Availability

Data transparency is maintained.

## Additional Points

*Centrifuge for Preparation of I-PRF.* Dentifuge LD C-10® Labtech Disposables, Ahmedabad 380015, Gujarat, India. *Marine Collagen Matrix.* Biofil sponge® Eucare Pharmaceuticals, Thirumudivakkam, Chennai 600044, Tamil Nadu, India.
